# Maternal-Infant Respiratory Syncytial Virus and Influenza A Virus Antibody Transfer in Preterm and Full-term Infants

**DOI:** 10.1093/ofid/ofae723

**Published:** 2024-12-30

**Authors:** Kalee E Rumfelt, Mindy Pike, Jennifer E Stolarczuk, Ava Lekander, Adam S Lauring, Linda O Eckert, Janet A Englund, Emily T Martin, Alisa B Kachikis

**Affiliations:** Department of Epidemiology, University of Michigan, Ann Arbor, Michigan, USA; Department of Obstetrics and Gynecology, University of Washington, Seattle, Washington, USA; Department of Obstetrics and Gynecology, University of Washington, Seattle, Washington, USA; School of Medicine, University of Washington, Seattle, Washington, USA; Division of Infectious Diseases, Department of Internal Medicine, University of Michigan, Ann Arbor, Michigan, USA; Department of Microbiology and Immunology, University of Michigan, Ann Arbor, Michigan, USA; Department of Obstetrics and Gynecology, University of Washington, Seattle, Washington, USA; Department of Global Health, University of Washington, Seattle, Washington, USA; Department of Pediatrics, Seattle Children's Hospital Research Institute, Seattle, Washington, USA; Department of Pediatrics, University of Washington, Seattle, Washington, USA; Department of Epidemiology, University of Michigan, Ann Arbor, Michigan, USA; Department of Obstetrics and Gynecology, University of Washington, Seattle, Washington, USA

**Keywords:** antibody, IAV, RSV, transplacental, vaccination

## Abstract

**Background:**

Immunization against influenza and respiratory syncytial virus (RSV) protects pregnant individuals and their infants against infection via transplacental transport of immunoglobulin G (IgG). We sought to evaluate the quantity and efficiency of maternal influenza- and RSV-specific IgG transfer in pregnancies with preterm and full-term deliveries.

**Methods:**

Delivery samples from 115 maternal-infant pairs (2018-2021) were analyzed for RSV prefusion F and IAV-H3 and IAV-H1 antibodies using electrochemiluminescence assays. We used Wilcoxon rank sum tests, *t* tests, Pearson correlation coefficients (PCCs), and linear regression to evaluate distributions of IgG results by maternal influenza vaccination status and preterm birth (<37 weeks).

**Results:**

Approximately 70% of pregnant persons received influenza vaccine. Maternal and cord antibody concentrations were highest in the influenza-vaccinated group for IAV-H3 and IAV-H1 regardless of preterm birth status (maternal H3, *P* = .004; cord H3, *P* = .03; maternal H1, *P* = .0001; cord H1, *P* = .0002). Preterm infants had significantly lower cord to maternal IgG transfer ratios for IAV-H3 and RSV when compared with full-term infants (*P* ≤ .05). Correlations between maternal and cord IgG concentrations were significant (*P* ≤ .001) for all 3 viruses, with the strongest correlation for H3 (PCC: IAV-H3, 0.77; IAV-H1, 0.68; RSV, 0.62). Associations between maternal IgG transfer and preterm birth were significant for IAV-H3 and RSV (IAV-H3, β = −0.42; RSV, β = −0.63; *P* ≤ .05).

**Conclusions:**

Maternal antibody following vaccination or infection is readily transferred across the placenta. Preterm infants have higher influenza IgG following maternal influenza vaccination and are at highest risk of lower IgG transfer ratios without vaccination.

Influenza virus and respiratory syncytial virus (RSV) are major causes of seasonal respiratory infections and disproportionately affect young children [1, 2]. Of all deaths that occur in children <6 months of age, 3.6% can be attributed to RSV infection globally [1]. The burden of influenza disease is highest in infants and the elderly, with hospitalization for lower respiratory tract involvement resulting in high rates of morbidity and mortality [3]. Increased risk of severe infections from influenza and RSV in children aged <6 months as compared with their mothers is likely due to their developing immune systems, respiratory epithelia, smaller airways, as well as lower levels of preexisting antibodies from prior infections [4].

Respiratory virus vaccines administered to pregnant persons are effective in reducing infections and infection-related adverse events in these individuals and their infants [4–7]. Meta-analyses have estimated that, on average, the seasonal influenza vaccine is 63% and 48% effective at reducing the risk of laboratory-confirmed influenza in pregnant individuals and their infants <6 months old, respectively [8, 9]. The newly approved RSV bivalent prefusion F (preF) protein vaccine during pregnancy has been estimated to reduce the risk of RSV-associated severe medically attended lower respiratory infection in infants by approximately 80% during the first 6 months of life [10].

Influenza and RSV vaccines increase maternal concentrations of virus-specific antibodies and subsequently lead to active transplacental transfer of maternal immunoglobulin G (IgG) to the fetus in utero. IgG against the influenza hemagglutinin (HA) and the RSV preF protein can be measured to determine potential protective levels for influenza and RSV, respectively, although no widely accepted correlate of protection for either influenza or RSV currently exists [6, 7]. Although maternal influenza vaccination has been shown to decrease the risk of influenza infection, there is little information available surrounding the magnitude of transplacental antibody transfer after maternal influenza vaccination in observational settings [11]. It is important to understand factors that influence transplacental transfer of maternal antibody to introduce innovations and interventions to decrease risks of severe infection. In this study, we assessed transplacental IgG transfer of RSV preF, influenza A virus (IAV)–H3, and IAV-H1 and those with and without preterm delivery at <37 weeks’ gestational age, in paired maternal-cord delivery blood to identify factors associated with efficient transplacental IgG transfer.

## METHODS

### Participants

We recruited participants from a prospective cohort study investigating maternal immunizations in low- and high-risk pregnancies at the University of Washington (UW). Inclusion criteria were ability to obtain informed consent, singleton pregnancy, and availability of paired maternal-cord blood samples. Exclusion criteria were multiple pregnancy (eg, twin), known fetal or neonatal genetic anomaly, or small-for-gestational-age birth weight infants (<10th percentile for gestational age per Olsen growth curves) [12]. This study was reviewed and received ethics approval through the UW Human Subjects Division. All participants provided written informed consent.

### Variables

Clinical health, immunization, race and ethnicity, and insurance data were abstracted from electronic medical records and linked Washington State Immunization Registry data as previously described [13]. We considered insurance status categories as public, private, Tricare (military), federal, or other. We calculated body mass index using maternal weight at the time of delivery. We categorized pregestational diabetes to participants with type 1 or 2 diabetes mellitus and defined chronic hypertension as participants diagnosed with hypertension before 20 weeks’ gestational age. We defined preeclampsia with or without severe features, chronic hypertension with superimposed preeclampsia, or eclampsia based on American College of Obstetricians and Gynecologists' definitions [14]. We categorized autoimmune or inflammatory conditions as participants with conditions including systemic lupus erythematous or Crohn disease, respectively. We considered participants as being on immunosuppressing medications if they received long-term corticosteroids, biologics, or other immunosuppressants during pregnancy (Table 1). We categorized birth quarter by dividing a year into 4 sections: January to March, April to June, July to September, and October to December. We defined low birth weight deliveries as infants born weighing <2500 g.

**Table 1.** Demographic and Baseline Characteristics

	No. (%) or Median (IQR)			
	Total (N = 115)	PTB <37 wk (n = 29)	FTB ≥37 wk (n = 86)	*P* Value^a^
Enrollment year				.19
2018	18 (15.7)	3 (10.3)	15 (17.4)	
2019	17 (14.8)	8 (27.6)	9 (10.5)	
2020	46 (40.0)	10 (34.5)	36 (41.9)	
2021	34 (29.6)	8 (27.6)	26 (30.2)	
Maternal age, y	33 (30–37)	31 (28–38)	33 (30–37)	.37
Gravidity	2 (1–4)	2 (1–4)	2 (1–3)	.44
Parity	0 (0–1)	0 (0–1)	1 (0–1)	.18
Race^b^				.20
American Indian or Alaska Native	1 (0.9)	1 (3.4)	0 (0.0)	
Pacific Islander	3 (2.6)	2 (6.9)	1 (1.2)	
Asian	16 (13.9)	3 (10.3)	13 (15.1)	
Black or African American	5 (4.4)	2 (6.9)	3 (3.5)	
White	83 (72.2)	20 (69.1)	63 (73.3)	
Other	7 (6.1)	1 (3.4)	6 (7.0)	
Hispanic ethnicity	12 (10.4)	3 (10.3)	9 (10.5)	.63
Insurance status^b^				.03
Public	19 (16.5)	9 (31.0)	10 (11.6)	
Private	91 (79.1)	18 (62.1)	73 (84.9)	
Tricare/federal/other	5 (4.4)	2 (6.9)	3 (3.5)	
Body mass index at delivery	31.2 (27.1–34.2)	29.7 (27.0–34.4)	31.5 (27.7–34.0)	.56
Pregestational diabetes mellitus	2 (1.7)	2 (6.9)	0 (0.0)	.06
Preeclampsia	6 (5.2)	4 (13.8)	2 (2.3)	.04
Chronic hypertension	10 (8.7)	4 (13.8)	6 (7.0)	.22
Autoimmune/inflammatory disorder^c^	3 (2.6)	1 (3.4)	2 (2.3)	.59
Immunosuppressing medications^d^	3 (2.6)	1 (3.4)	2 (2.3)	.59
Vaccine receipt during pregnancy				
Influenza	83 (72.2)	23 (79.3)	60 (69.8)	.32
Tdap	109 (94.8)	25 (86.2)	84 (97.7)	.02

Abbreviations: FTB, full-term birth; PTB, preterm birth; Tdap, tetanus, diphtheria, acellular pertussis.

^a^Significant differences between PTB and FTB were assessed for continuous variables by *t* test and categorical variables by chi-square tests and Fisher exact tests.

^b^Race and insurance missing data were <10% and collapsed into the other category.

^c^Autoimmune and inflammatory disorders include systemic lupus erythematous or Crohn disease.

^d^Immunosuppressing medications include long-term corticosteroid use, biologics, or other medications.

### Antibody Testing

Maternal blood samples were collected within 72 hours of delivery and cord blood samples at delivery. Blood samples were centrifuged at 1800 rpm for 20 minutes and sera stored at −80 °C. Maternal total IgG testing was performed by the UW Immunology Clinical Laboratory on maternal and cord serum samples using an Optilite analyzer with standard reagents. Maternal and cord sera were tested in parallel on the same day for IgG against RSV preF, IAV A/Hong Kong/4801/2014 HA (H3), and A/Michigan/45/2015 HA (H1) with an electrochemiluminescence immunoassay (Meso Scale Discovery [MSD]). Information regarding seasonal influenza vaccine strains and match to MSD antigens is presented in the supplement (Supplementary Table 1) [15]. Serum samples were diluted 1:5000 and 1:25 000 and processed according to the manufacturer's protocol [16]. Quantification of specimen antibody (arbitrary units per milliliter) was determined by plotting assay outputs onto the log-transformed standard curve generated from serially diluted calibrators. We defined efficient maternal antibody transfer as a cord to maternal (cord:maternal) antibody ratio >1.

### Statistical Analysis

Baseline demographic and pregnancy characteristics were described, and these variables were compared by *t* tests, chi-square tests, and Fisher exact tests for comparisons with small numbers. We categorized pregnancies into those with preterm deliveries (gestational age <37 weeks) and those with full-term deliveries (≥37 weeks). We evaluated the relationship between preterm birth (PTB) and maternal and cord RSV and IAV IgG levels using Wilcoxon rank sum tests. We assessed this relationship using log-transformed maternal and cord RSV and IAV IgG levels with linear regression. Similar analyses were performed for untransformed ratios of infant to maternal RSV and IAV IgG, which we tested with *t* tests and linear regression. We assessed the correlation between infant and maternal RSV and IAV IgG for the entire sample and across PTB status. We evaluated the relationship between participants with and without influenza vaccination during pregnancy and maternal and cord IAV IgG levels using Wilcoxon rank sum tests stratified by birth status. We also compared cord:maternal IAV untransformed IgG ratios between pairs with and without maternal influenza vaccination using *t* tests. We assessed statistical differences of maternal IgG concentrations, cord IgG concentrations, and cord:maternal IgG transfer ratio between maternal vaccinations that occurred >6 and <6 months before, >3 and <3 months before, and >1 and <1 month before delivery using *t* tests. Covariates were selected a priori and based on significant associations between the exposure of PTB and the outcomes of RSV and IAV cord IgG. The first minimally adjusted linear regression model included the annual quarter of the infant's birth date. A second minimally adjusted linear regression model included annual birth quarter and insurance status. We performed statistical analyses using SAS software version 9.4 (SAS Institute Inc) and considered a 2-sided *P* < .05 to be statistically significant [17]. We followed STROBE guidelines (Strengthening the Reporting of Observational Studies in Epidemiology) [18].

## RESULTS

### Baseline Characteristics and Pregnancy Outcomes

Between June 2018 and July 2021, 115 maternal-infant pairs met inclusion criteria. Most births (69.6%) occurred in 2020 to 2021. Of 115 infants, 29 (25.2%) were born preterm. Demographic and baseline medical information was similar among preterm and full-term pregnancies (Table 1), with the exception of insurance status, preeclampsia, and receipt of Tdap vaccine (tetanus, diphtheria, acellular pertussis) during pregnancy. A large proportion of participants in this cohort were vaccinated with the annual influenza vaccine (72.2%) and received the Tdap vaccine (94.8%) during pregnancy. Birth dates in this cohort were relatively evenly distributed across birth quarters, with the greatest number of infants born in quarter 3 (33.9%) and the fewest born in quarter 2 (19.1%; Table 2). As expected, birth weight had a significantly lower median in preterm infants (*P* < .001). A small subset of infants was born low birth weight (14.8%) and admitted to the neonatal intensive care unit (21.1%).

**Table 2.** Pregnancy Outcomes

	No. (%) or Median (IQR)			
	Total (n = 115)	PTB <37 wk (n = 29)	FTB ≥37 wk (n = 86)	*P* Value^a^
Birth quarter				.17
Q1: January–March	28 (24.4)	11 (37.9)	17 (19.8)	
Q2: April–June	22 (19.1)	6 (20.7)	16 (18.6)	
Q3: July–September	39 (33.9)	6 (20.7)	33 (38.3)	
Q4: October–December	26 (22.6)	6 (20.7)	20 (23.3)	
Gestational age at delivery, wk	39.0 (36.9–39.6)	35.4 (33.9 36.00)	39.1 (39.0–39.9)	<.001
Range	26.0–41.9	26.0–36.9	37.00–41.9	
Low birth weight delivery, <2500 g	17 (14.8)	16 (55.2)	1 (1.2)	<.001
Mode of delivery				.23
Vaginal delivery	55 (47.8)	10 (34.5)	45 (52.3)	
Cesarean section	60 (52.2)	19 (65.5)	41 (47.7)	
Birth weight, g	3291 (2292–3561)	2460 (1969–3116)	3352 (3188–3651)	<.001
Sex				.13
Female	64 (55.7)	13 (44.8)	51 (59.3)	
Male	51 (44.3)	16 (55.2)	35 (40.7)	
NICU admission	24 (21.1)	21 (72.4)	3 (3.5)	<.001
Maternal total IgG at delivery	691 (600–822)	770 (491–841)	681 (603–818)	.62

Abbreviations: FTB, full-term birth; IgG, immunoglobulin G; NICU, neonatal intensive care unit; PTB, preterm birth.

^a^Significant differences between PTB and FTB were assessed for continuous variables by *t* test and categorical variables by chi-square tests and Fisher exact tests.

### IgG Analyses

Higher maternal and cord IgG antibody levels were seen for RSV, IAV-H3, and IAV-H1 in full-term as compared with preterm infants. The median transfer ratio of IgG was highest for RSV in the total cohort and the FTB group, while the median transfer ratio was highest in the PTB group for IAV-H3. When participants were stratified by maternal influenza vaccination and birth status, the highest median maternal and cord IgG concentrations were in the vaccinated FTB group and the lowest in the unvaccinated PTB group for IAV-H3 and IAV-H1 (Table 3). Maternal and cord antibody concentrations were highest in the influenza-vaccinated group for IAV-H3 and IAV-H1 regardless of PTB status (maternal H3, *P* = .004; cord H3, *P* = .03; maternal H1, *P* = .0001; cord H1, *P* = .0002; not shown). The median IAV-H3 IgG transfer ratio was highest in the unvaccinated group for PTB and FTB, but the difference was not significant. The differences in maternal and cord IgG concentrations between vaccinated and unvaccinated mothers were not significantly different by birth year or when vaccination was stratified by <6, <3, or <1 month prior to delivery (not shown).

**Table 3.** Maternal and Cord IAV Anti-H3 and Anti-H1 Protein Results in the General Cohort and by Preterm and Term Deliveries Stratified by Annual Influenza Vaccination During Pregnancy

	No. or Median (IQR)			
	Total	Vaccinated	Unvaccinated	*P* Value
**Anti-H3 IgG**				
PTB^a^	29	23	6	
Maternal	24 284 (11 019–55 226)	30 443 (15 923–76 014)	12 549 (6871–16 212)	.01
Cord	26 534 (13 977–49 613)	35 711 (18 782–52 588)	14 847 (10 915–22 556)	.02
Ratio	1.19 (0.88–1.41)	1.08 (0.85–1.39)	1.36 (1.18–1.59)	.67
FTB^b^	86	60	26	
Maternal	29 472 (14 144–51 345)	34 906 (17 190–57 792)	25 575 (9772–40 686)	.05
Cord	49 094 (24 237–82 604)	54 229 (30 000–97 968)	39 695 (17 005–61 066)	.08
Ratio	1.61 (1.35–1.95)	1.60 (1.35–1.95)	1.65 (1.28–1.96)	.78
**Anti-H1 IgG**				
PTB^c^	29	23	6	
Maternal	34 265 (14 133–100 847)	45 121 (27 850–351 427)	6827 (5203–23 413)	.004
Cord	39 164 (26 581–86 775)	51 202 (26 784–103 483)	10 246 (5646–32 185)	.004
Ratio	1.08 (0.81–1.49)	1.10 (0.66–1.50)	1.23 (0.99–1.47)	.92
FTB^d^	86	60	26	
Maternal	37 496 (19 271–103 467)	51 508 (31 762–123 346)	23 517 (6967–51 996)	.004
Cord	59 625 (31 913–161 903)	81 359 (41 697–197 564)	39 822 (11 708–77 774)	.003
Ratio	1.64 (1.18–1.96)	1.65 (1.30–1.90)	1.60 (1.13–2.02)	.49

Statistical differences between vaccinated and unvaccinated mothers were assessed for maternal and cord anti-spike IgG by Wilcoxon rank sum test. The ratio is calculated as cord IgG divided by maternal IgG, and statistical differences between vaccinated and unvaccinated mothers were assessed by *t* test.

Abbreviations: FTB, full-term birth; IAV, influenza A virus; IgG, immunoglobulin G; PTB, preterm birth.

^a^Maternal and cord IAV A/Hong Kong/4801/2014 anti-H3 IgG results in pregnancies with PTB (<37 weeks’ gestation).

^b^Maternal and cord IAV A/Hong Kong/4801/2014 anti-H3 IgG results in pregnancies with FTB (≥37 weeks’ gestation).

^c^Maternal and cord IAV A/Michigan/45/2015 anti-H1 IgG results in pregnancies with PTB (<37 weeks’ gestation).

^d^Maternal and cord IAV A/Michigan/45/2015 anti-H1 IgG results in pregnancies with FTB (≥37 weeks’ gestation) stratified by annual influenza vaccination during pregnancy.

**Figure 1.**
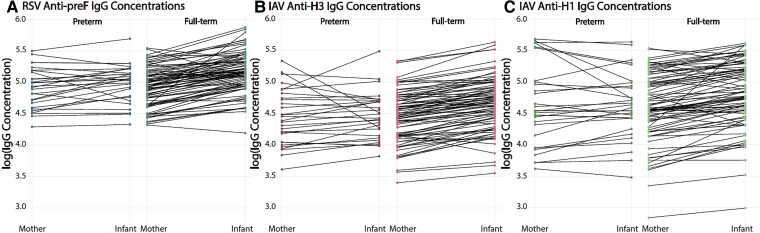
Paired profiles of maternal and cord IgG concentrations to illustrate transfer by preterm birth and full-term birth. *A-C*, Visualization of maternal antibody transfer for each mother-infant pair by log-adjusted IgG across prematurity: RSV anti-preF, IAV anti-H3, and IAV anti-H1. H1, A/Michigan/45/2015 HA (H1); H3, A/Hong Kong/4801/2014 HA (H3); IAV, influenza A virus; IgG, immunoglobulin G; preF, prefusion F; RSV, respiratory syncytial virus.

**Figure 2.**
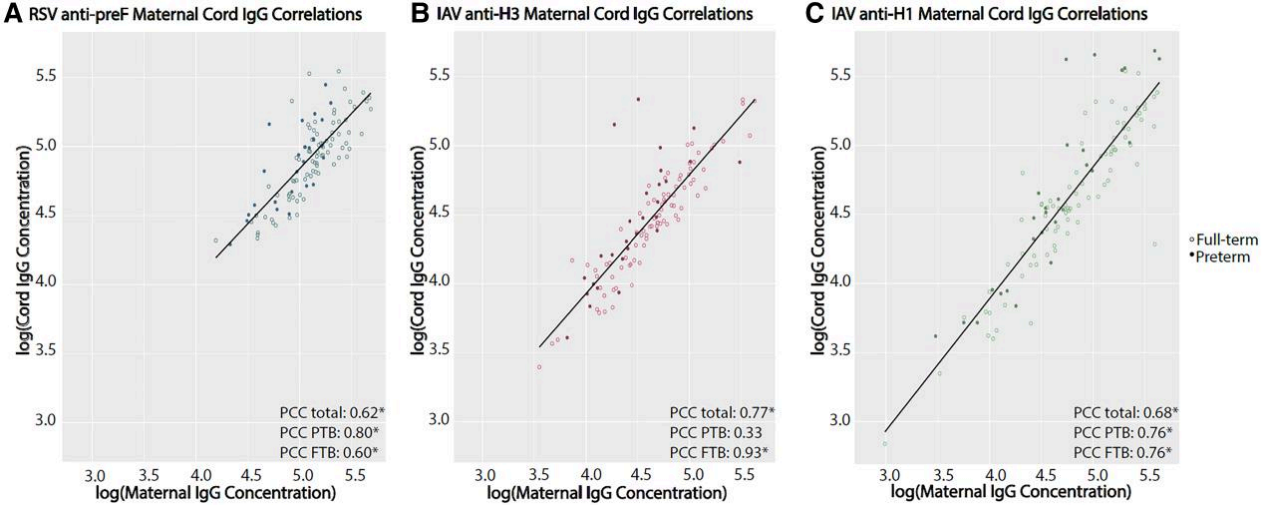
Correlations between maternal and cord IgG concentrations by preterm birth and full-term birth. *A-C*, RSV anti-preF, IAV anti-H3, and IAV anti-H1. FTB, full-term birth; H1, A/Michigan/45/2015 HA (H1); H3, A/Hong Kong/4801/2014 HA (H3); IAV, influenza A virus; IgG, immunoglobulin G; PCC, Pearson correlation coefficient; preF, prefusion F; PTB, preterm birth; RSV, respiratory syncytial virus.

When PTB and FTB were compared without stratification by maternal vaccination, median maternal IgG level, cord IgG level, and transfer ratio were significantly lower in the PTB group for all specific antibodies, including RSV anti-preF IgG cord concentration and IgG transfer ratio and IAV-H3 IgG cord concentration and IgG transfer ratio (Table 4, Figure 1). Maternal and cord concentrations were moderately correlated with each other for RSV, IAV-H3, and IAV-H1 (Pearson correlation coefficient [PCC]: RSV, 0.62; IAV-H3, 0.77; IAV-H1, 0.68; *P* < .0001). The correlations for full-term infants and their mothers were similar for RSV and higher for IAV-H3 and IAV-H1 as compared with the correlations in the total sample (full-term PCC: RSV, 0.60; IAV-H3, 0.93; IAV-H1, 0.76; *P* < .0001). The correlation remained significant and increased for RSV and IAV-H1 in the PTB group (PCC: RSV, 0.80; IAV-H1, 0.76; *P* < .0001) but decreased and was not significant for IAV-H3 (PCC: IAV-H3, 0.33; *P* = .09; Figure 2).

**Table 4.** Maternal and Cord RSV Anti-preF, IAV Anti-H3, and IAV Anti-H1 Protein Results in the General Cohort and by PTB and FTB Status

	Median (IQR)			
	Total (N = 115)	<37 wk (n = 29)	≥37 wk (n = 86)	*P* Value
Anti-preF IgG^a^				
Maternal	87 052 (46 999–144 508)	87 052 (46 999–145 311)	88 703 (51 379–136 642)	.84
Cord	135 652 (86 280–207 152)	112 113 (60 468–139 984)	146 182 (93 478–215 557)	.01
Ratio	1.64 (1.17–2.02)	1.14 (0.98–1.57)	1.75 (1.37–2.09)	.03
Anti-H3 IgG^b^				
Maternal	28 565 (14 144–53 089)	24 284 (11 019–55 226)	29 472 (14 144–51 345)	.73
Cord	43 361 (19 534–74 230)	26 534 (13 977–49 613)	49 094 (24 237–82 604)	.01
Ratio	1.50 (1.18–1.93)	1.19 (0.88–1.41)	1.61 (1.35–1.95)	.001
Anti-H1 IgG^c^				
Maternal	37 134 (18 782–103 467)	34 265 (14 133–100 847)	37 496 (19 271–103 467)	.81
Cord	56 545 (26 937–156 580)	39 164 (26 581–86 775)	59 625 (31 913–161 903)	.08
Ratio	1.49 (1.08–1.80)	1.08 (0.81–1.49)	1.64 (1.18–1.96)	.09

Statistical differences between vaccinated and unvaccinated mothers were assessed for maternal and cord anti-spike IgG by Wilcoxon rank sum test. Ratio is calculated as cord IgG divided by maternal IgG, and statistical differences between vaccinated and unvaccinated mothers were assessed by *t* test.

Abbreviations: FTB, full-term birth; IAV, influenza A virus; IgG, immunoglobulin G; PTB, preterm birth; preF, prefusion F; RSV, respiratory syncytial virus.

^a^Maternal and cord RSV anti-preF IgG results in the entire cohort and by PTB and FTB status.

^b^Maternal and cord IAV A/Hong Kong/4801/2014 anti-H3 IgG results in the entire cohort and by PTB and FTB status.

^c^Maternal and cord IAV A/Michigan/45/2015 anti-H1 IgG results in the entire cohort and by PTB and FTB status.

As expected, PTB was not significantly associated with log-transformed maternal IgG concentrations for any of the virus-specific antibodies in either the null model or when adjusted for birth quarter (Table 5). PTB was associated with significant decreases in log-transformed cord IgG RSV and IAV antibody concentrations before and after adjustment; PTB was also significantly associated with a substantial decrease in cord:maternal IgG transfer ratios for RSV and IAV-H3 (47% and 34% decrease, respectively). Results for a further adjusted model including insurance status produced similar results to the adjusted models presented in Table 5 with slight decreases in precision; therefore, these results were not reported.

**Table 5.** Associations Between Preterm Birth and Maternal and Cord RSV Anti-preF, IAV-H3, and IAV-H1 IgG

	RSV Anti-preF				IAV Anti-H3				IAV Anti-H1			
IgG	β (95% CI)	*P* Value	β^a^ (95% CI)	*P* Value	β (95% CI)	*P* Value	β^a^ (95% CI)	*P* Value	β (95% CI)	*P* Value	β^a^ (95% CI)	*P* Value
Maternal												
PTB <37 wk	−0.02 (−.15, .11)	.81	−0.03 (−.16, .02)	.65	−0.02 (−.19, .15)	.82	−0.03 (−.20, .15)	.76	0.01 (−.23, .25)	.91	0.02 (−.22, .27)	.85
Cord												
PTB <37 wk	−0.17 (−.30, −.04)	.01	−0.17 (−.31, −.04)	.01	−0.20 (−.37, −.02)	.03	−0.20 (−.37, −.02)	.03	−0.018 (−.40, .04)	.11	−0.17 (−.39, .06)	.14
Cord:maternal ratio												
PTB <37 wk	−0.63 (−1.21, −.06)	.03	−0.59 (−1.17, −.01)	.05	−0.42 (−.66, −.17)	.001	−0.41 (−.66, −.16)	.002	−0.70 (−1.50, .10)	.09	−0.73 (−1.54, .09)	.08

Reference for this analysis was FTB.

Abbreviations: FTB, full-term birth; H1, A/Michigan/45/2015 HA (H1); H3, A/Hong Kong/4801/2014 HA (H3); IAV, influenza A virus; IgG, immunoglobulin G; PTB, preterm birth; preF, prefusion F; RSV, respiratory syncytial virus.

^a^Adjusted for birth quarter.

## DISCUSSION

We studied the transfer of maternal to infant RSV and influenza IgG antibodies and documented using standard and novel immunoassays to demonstrate not only the efficient transfer of these antibodies in preterm as well as full-term infants but also the fact that preterm infants can benefit from influenza immunization during pregnancy. We found that maternal and cord antibody concentrations were well correlated for RSV, IAV-H3, and IAV-H1. Importantly, we observed efficient transplacental transfer of RSV, IAV-H3, and IAV-H1 IgG antibodies in preterm as well as full-term infants. When we further stratified our analyses by maternal influenza vaccination, maternal and cord antibody concentrations were highest for IAV-H3 and IAV-H1 in the vaccinated groups regardless of gestational age category at delivery. However, cord antibody concentrations and cord:maternal IgG transfer ratios were significantly lower in the PTB group for RSV and IAV-H3. Associations between cord concentration and PTB as well as less efficient maternal IgG transfer ratios and PTB were significant (*P* ≤ .05) for RSV and IAV-H3. This demonstrates that influenza vaccination during pregnancy has the potential to enhance transplacental IgG transfer even for preterm infants, which is important given high rates of preterm delivery globally and increased risks of morbidity and mortality in preterm infants.

In our study, the median cord:maternal transfer ratio was very high: 1.64 for RSV, 1.50 for IAV-H3, and 1.49 for IAV-H1. These findings are similar to other studies investigating transplacental antibody transfer for common respiratory viruses, including RSV [19–21]. For example, Albrecht et al [21] calculated an average maternal-infant antibody transfer ratio of 1.5 for IAV but did not investigate differences between H3 and H1. A study of 57 full-term mother-infant pairs from Seattle calculated cord:maternal antibody transfer ratios of 1.15 for RSV, 1.22 for IAV-H3, and 1.38 for IAV-H1 [19]. While differences in effect size might be due to sampling site, vaccine exposure, and IgG detection methods, our findings confirm that RSV and influenza specific antibodies are transferred very efficiently across the placenta and in similar ratios.

We found that cord:maternal antibody transfer ratios were lower in pregnancies with preterm infants, a finding that is well established in the literature for multiple virus-specific antibodies [20, 21]. More recently, in studies including Alaska Native and Seattle-based mother-infant pairs by Chu et al [19] and an Australian indigenous population by Homaira et al [22], the authors found significantly lower cord:maternal antibody transfer ratios in preterm as compared with full-term infants for RSV but did not see significant differences between groups for IAV-H3 or H1. The differences observed for IAV may be affected by the timing of predominant subtype circulation, sampling site, respiratory virus season of study, and infection history.

For IAV-H3 and IAV-H1, we found higher maternal and cord antibody concentrations in vaccinated individuals and their infants regardless of PTB status, with the largest discrepancies produced in the PTB group for IAV-H1. Zhong et al [5] investigated the difference in H1N1 and H3N2 IgG concentrations in pregnant individuals and infants at birth and found significantly higher concentrations in those persons vaccinated during pregnancy and their infants for H1N1 but saw no significant differences in antibody concentration for H3N2. However, they limited their H3N2 analysis to the 2013–2014 and 2014–2015 influenza seasons, which in turn limited the sample size and power to detect significant differences.

It is important to consider that due to antigenic drift, antigens included in the annual influenza vaccine are often changed, and we used the same target strains for all specimens regardless of year for the MSD assay in this study. For example, the strains used in the MSD assay, A/Hong Kong/4801/2014 HA (H3) and A/Michigan/45/2015 HA (H1), matched the 2017–2018 season vaccine strains and the H1 2018–2019 season vaccine strain but did not match vaccine strains for subsequent seasons (Supplementary Table 1) [15]. This is an important distinction because the assay targets may not fully represent the true protection elicited by vaccination in each season. In turn, the MSD output gives a representation of partial or nonspecific protection elicited from vaccination after the 2017–2018 season. Although these results may not provide a true representation of the protection conferred from vaccination, it is reasonable to assume that vaccination may have boosted antibodies toward the MSD H3 and H1 targets, which could represent a cross-reactive correlate of protection and be considered a correlate of season-specific protection.

Strengths of our study include a moderately sized multiyear cohort with an adequate representation of pregnancies with preterm infants. This allowed us to investigate PTB as a risk factor for less efficient cord:maternal antibody transfer across multiple respiratory virus seasons. We also collected reliable vaccination data utilizing the electronic medical record linked to the Washington State Immunization Registry, providing access to reliable vaccination data. This study supports previous data showing that maternal influenza immunizations increase maternal and cord H3N2 and H1N1 IgG concentrations. This evidence has been primarily presented in clinical trials with little information known about this association in observational settings. This study helps fill this knowledge gap by presenting increased maternal and cord IAV IgG concentrations in an observational setting. Furthermore, laboratory methods used in this study utilized MSD, a novel, previously validated, high-throughput assay that requires a minimal amount of sera, limiting the time and resources needed to obtain meaningful results [16, 23].

Our study was limited by the fact that we were unable to document prior infection with influenza or RSV. Since prior infection may influence antibody concentrations more strongly in unvaccinated people, this may be especially relevant to our RSV analysis where all participants were unvaccinated [5]. Also, we did not follow participants and their infants for infections after birth [6]. This gap points toward future work that should be done to understand how vaccination timing and IgG concentrations in the mother and infant affect subsequent infection risk. These data can be used to make recommendations about the optimization of maternal influenza vaccination to potentially include protection of preterm infants. There is a possible detection issue from mismatched seasonal influenza vaccine strains and the influenza MSD antigens for the 2018–2019, 2019–2020, and 2020–2021 influenza seasons [15]. Although the vaccine strains and MSD antigens are different in these seasons, the comparisons are internally controlled for by assessing cord:maternal transfer ratio. This study period coincided with COVID-19 mitigation measures, which could have affected the generalizability of the last season of the study. However, upon further investigation there were not large differences in the distributions of maternal factors, birth outcomes, maternal IgG concentrations, cord IgG concentrations, or cord:maternal IgG transfer ratios between the periods before and after COVID-19 mitigation measures. Last, we were unable to investigate biological variation or antibody function in this study. Therefore, we cannot draw any conclusions to whether biologic variability influences antibody concentrations or how functional the measured antibodies were.

Our results demonstrate that cord antibody concentrations are higher in vaccinated individuals in full-term and preterm infants, illustrating that maternal influenza vaccination is an important mitigation technique to boost infant antibody concentration and potentially decrease the risk of infection in infants, particularly infants born preterm. Additionally, we saw cord:maternal transfer ratios >1 for RSV and influenza in unvaccinated pregnancies, indicating that efficient antibody transfer can occur following natural infection. Infants born preterm are more likely to have lower cord antibody concentrations and less efficient maternal antibody transfer for RSV and IAV-H3, putting them at a greater risk for infection after birth. Our study also acts as a baseline for comparison with data after maternal RSV vaccine uptake increases and potential assistance in validating correlates of protection against RSV infection in the future.

## Supplementary Material

ofae723_Supplementary_Data

## Supplementary Data

Supplementary materials are available at *Open Forum Infectious Diseases* online. Consisting of data provided by the authors to benefit the reader, the posted materials are not copyedited and are the sole responsibility of the authors, so questions or comments should be addressed to the corresponding author.

## Notes

***Acknowledgments.*** We thank the participating individuals who contributed their valuable time and samples to this study. We also express our gratitude to the clinical care teams on UW Medicine Center Montlake's Labor and Delivery and Mother Baby Units and Northwest's Childbirth Center who made this research study possible.

***Author contributions.*** K. E. R. performed all the laboratory testing, helped develop the research plan, performed statistical analysis, wrote portions of the manuscript, created the tables and figures, and assisted in the editing process. M. P. cleaned and managed the data, consulted on the statistical analysis, wrote portions of the manuscript, and assisted in the editing process. J. E. S. entered and cleaned data and assisted with study methodology and procedures. A. L. entered and cleaned data and assisted with study methodology and procedures. A. S. L. assisted in the editing process. L. O. E. assisted in the editing process. J. A. E. developed the research plan, wrote portions of the manuscript, and assisted in the editing process. E. T. M. helped develop the research plan and assisted in the editing process. A. B. K. developed the research plan, wrote portions of the manuscript, and assisted in the editing process.

***Conference presentations.*** Individual and Population Immunity Toward Respiratory Viruses, Hong Kong, November 2023. ReSViNET, Mumbai, India, February 2024. INMIS, San Jose, Costa Rica, March 2024.

***Patient consent statement.*** Informed written consent was obtained from each patient prior to participating in the study. This study received ethics approval from the Seattle Children’s Hospital Institutional Review Board and the University of Washington Human Subjects Division.

***Financial support.*** This work was supported by the National Institute of Allergy and Infectious Diseases (K23 AI153390 to A. B. K.); a Women's Reproductive Health Research award through the Eunice Kennedy Shriver National Institute for Child Health and Human Development (2 K12HD001264-21 to A. B. K.); the Thrasher Research Fund (to A. B. K.); and the National Center for Advancing Translational Sciences of the National Institutes of Health (UL1 TR002319 to A. B. K.).
