# Supplementary material for: Maternal-Infant Respiratory Syncytial Virus and Influenza A Virus Antibody Transfer in Preterm and Full-term Infants
Source: Open Forum Infect Dis. 2024 Dec 30;12(1):ofae723. doi: 10.1093/ofid/ofae723 (PMC11697169; doi:10.1093/ofid/ofae723)
Supplement: ofae723_Supplementary_Data [file ofae723_supplementary_data.docx]

**Supplemental Table 1. Seasonal IAV vaccine strains and MSD IAV antigens**

| **Season** | **H3 Vaccine Strain** | **H3 MSD Antigen** | **H1 Vaccine Strain** | **H1 MSD Antigen** |
| --- | --- | --- | --- | --- |
| 2017-18 | **A/Hong Kong/4801/2014** | **A/Hong Kong/4801/2014** | **A/Michigan/45/2015** | **A/Michigan/45/2015** |
| 2018-19 | A/Singapore/INFIMH-16-0019/2016 | A/Hong Kong/4801/2014 | **A/Michigan/45/2015** | **A/Michigan/45/2015** |
| 2019-20 | A/Kansas/14/2017 | A/Hong Kong/4801/2014 | A/Brisbane/02/2018 | A/Michigan/45/2015 |
| 2020-21 | A/Hong Kong/2671/2019 | A/Hong Kong/4801/2014 | A/Guangdong-Maonan/SWL1536/2019 | A/Michigan/45/2015 |
| NOTES: Bold text indicates matching vaccine strain and MSD antigen | | | | |
